# Research on BOLD-fMRI Data Denoising Based on Bayesian Estimation and Adaptive Wavelet Threshold

**DOI:** 10.1155/2021/8819384

**Published:** 2021-02-05

**Authors:** Zini Jian, Xianpei Wang, Xueting Liu, Meng Tian, Quande Wang, Jiangxi Xiao

**Affiliations:** ^1^Electronic Information School, Wuhan University, Wuhan 430072, China; ^2^Peking University First Hospital, Beijing 100034, China

## Abstract

The acquisition of functional magnetic resonance imaging (fMRI) images of blood oxygen level-dependent (BOLD) effect and the signals to be analyzed is based on weak changes in the magnetic field caused by small changes in blood oxygen physiological levels, which are weak signals and complex in noise. In order to model and analyze the pathological and hemodynamic parameters of BOLD-fMRI images effectively, it is urgent to use effective signal analysis techniques to reduce the interference of noise and artifacts. In this paper, the noise characteristics of functional magnetic resonance imaging and the traditional signal denoising methods are analyzed. The Bayesian decision criterion takes into account the probability of the total occurrence of all kinds of references and the loss caused by misjudgment and has strong discriminability. So, an improved adaptive wavelet threshold denoising method based on Bayesian estimation is proposed. By using the correlation characteristics of multiscale wavelet coefficients, the corresponding wavelet components of useful signals and noises are processed differently; while retaining useful frequency information, the noise is weakened to the greatest extent. The new adaptive threshold wavelet denoising method based on Bayesian estimation is applied to the actual experiment, and the results of OEF (oxygen extraction fraction) are optimized. A series of simulation experiments are carried out to verify the effectiveness of the proposed method.

## 1. Introduction

OEF (oxygen extraction fraction) refers to the ratio of oxygen uptake from the blood to the total oxygen content of arterial blood when oxygen-rich arterial blood flow passes through capillaries in a region of the brain, which reflects the activity of oxygen metabolism in the brain [1]. It is one of the three physiological indicators of energy metabolism in brain tissue, along with CBF (cerebral blood flow) and CMRO2 (cerebral metabolic rate of oxygen) [2]. OEF is also the difference in oxygen content between arterial and venous blood when tissues take oxygen from the capillary network for utilization. OEF indicates the ability of neurons to utilize oxygen and is an indicator of energy metabolism in brain tissue. Under normal circumstances, the metabolic oxygen supply and oxygen demand of neurons in the brain are in a dynamic equilibrium. When some abnormal conditions such as ischemic stroke and brain injury destroy this equilibrium relationship, physiological metabolic indicators such as cerebral blood flow and OEF will be abnormal. Therefore, the measurement of OEF is of great significance for the prevention and treatment of ischemic stroke. Over the past 25 years, the increase of OEF has been recognized as the gold standard to prove the existence of cerebral hemodynamic damage [3]. In addition, there is no significant difference in OEF values between gray matter and white matter, and the variability in the population is small, which is suitable as a measurement index. At present, the relatively mature OEF measurement technology is to measure the cerebral hemodynamic parameters through PET (positron emission tomography) technology [4]. However, PET technology is very expensive, and it also needs to use ^15^O_2_ as a radioactive labeling substance. The radioactive substance itself has radiation trauma effect on the tested human body, and the half-life of this radioactive substance is very short, only about 2 minutes, resulting in this technology not being easy to apply in the clinic. With the rapid development of magnetic resonance imaging (MRI) and blood oxygen level-dependent (BOLD) effects, more and more studies have begun to use BOLD for T2∗-weighted imaging to study and process cerebral blood perfusion and metabolism [5–8]. The difference between this method and other imaging methods is that MRI has the highest spatial resolution for brain lesions and is safer and more comprehensive in tissue structure, physiology, function, metabolism, and other aspects, especially in the diagnosis of neurological diseases.

In 1994, Haacke et al. [9] proposed a theoretical model to measure the signal decline caused by extracellular deoxygenated hemoglobin through a new MRI sequence. In 2000, An and Lin [10] proposed an MRI sequence that combined GRE and spin echo (SE) and solved the brain tissue OEF value through a mathematical model. In 2003, An and Lin continued to improve and reorganize the MRI sequence, trying to measure the OEF value from another perspective [11]. The sequence they proposed does not need to calculate *R*_2_, which can reduce the measurement error to a certain extent. Based on the research of An et al., researchers have developed a lot of OEF postprocessing software, which can directly output OEF measurement results after inputting the nuclear magnetic resonance images of GESSE sequences [12]. However, there are still few researches on measuring OEF with new nuclear magnetic resonance sequences or algorithms [13].

A mathematical model is established by using the functional magnetic resonance imaging of blood oxygen level-dependent (BOLD-fMRI) effect, which can accurately measure the OEF value of the human brain [14]. However, since the image signal is based on a small change in the physiological level of blood oxygen, the magnetic field is weakly changed, and the noise is complicated. So it is urgent to have effective signal analysis technology to reduce the interference of noise and artifacts [15, 16] and improve the accuracy of OEF measurement. In this paper, an adaptive wavelet threshold denoising method based on Bayesian estimation is proposed on the basis of traditional wavelet denoising. After the wavelet decomposition, according to the different wavelet subband characteristics, the improved wavelet scaling function parameter equation is used to determine the optimal threshold for each wavelet decomposition scale level. The simulation results show that compared with the traditional wavelet hard threshold and soft threshold denoising, this method can better improve the SNR and reduce the mean square error of the denoising image and has better denoising effect, making the final measurement result of OEF more accurate.

After reconstruction, the NMR image signal is inherently complex. Two signals are collected through the receiving coil. After orthogonal detection, the two image signals have zero mean value with equal variance and Gaussian noise except the phase difference. Magnetic resonance image signals obey Gaussian distribution in the signal region with high SNR and Rayleigh distribution in the signal region with low SNR [17]. The noise is no longer completely independent but related to the signal.

**Figure 1:**
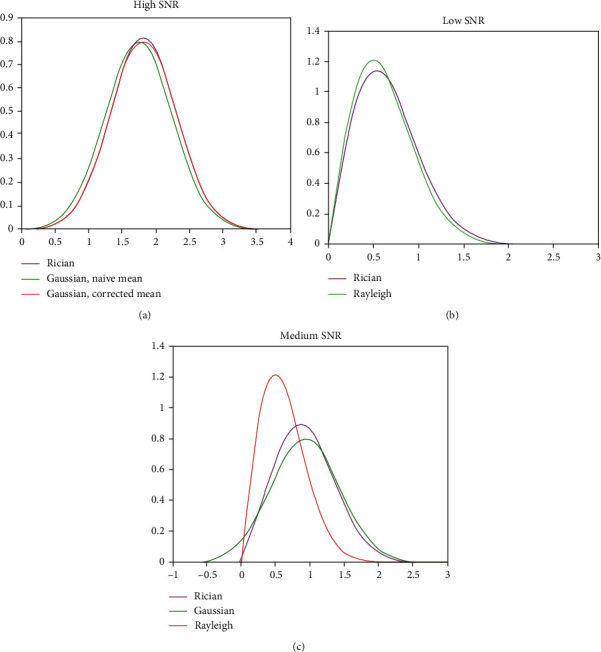
(a) When the SNR is high, the Rician distribution is close to the Gaussian distribution. (b) When the SNR is very low, the Rician distribution is close to the Rayleigh distribution. (c) When the SNR is medium, the Rician distribution is shown in the figure.

The noise in NMR image signals obeys Rician distribution, as shown in Figure 1.

As can be seen from the figure, if the signal-to-noise ratio is high, the Rician distribution [18] will be close to the Gaussian distribution; if the signal-to-noise ratio is close to 0 (in this case, only noise exists), the Rician distribution will become a Rayleigh distribution. When the signal-to-noise ratio is low, it is signal dependent, and it is very difficult to remove the random variation and deviation rate of Rician noise generation. How to choose an effective denoising method to effectively separate the signal from the noise is also very meaningful and challenging.

The wavelet threshold denoising method [19, 20] is to process the decomposed wavelet coefficients by finding appropriate thresholds in the appropriate method and to remove the wavelet coefficients belonging to the noise, leaving the wavelet coefficients belonging to the signal, and then reconstruct the processed wavelet coefficients to get the denoised signal. Classical threshold processing wavelet denoising has the following types: wavelet hard threshold denoising, soft threshold denoising, and soft and hard threshold denoising [21].

### 1.1. Wavelet Hard Threshold Denoising

**Figure 2:**
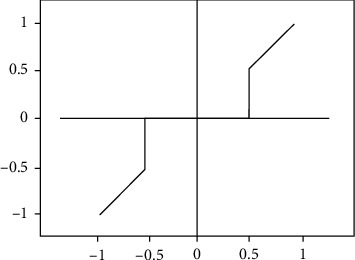
Wavelet hard threshold denoising.

As shown in Figure 2, the hard threshold denoising is that after the wavelet coefficients are decomposed, the wavelet coefficients with absolute values greater than *T* are retained, and the wavelet coefficients with absolute values less than *T* are set to zero. Set the threshold *T* to 0.5. The specific expression is as follows [22]:

(1) $$\begin{array}{c} \rho_{T}\left(x\right)=\left\{\begin{array}{cc} x, & \left|x\right|\geq T,\\ 0, & \left|x\right|\geq T, \end{array}\right. \end{array}$$

It can also be seen from the figure that the wavelet hard threshold denoising has a sudden change when the absolute value of the wavelet coefficient is equal to *T*, which will lead to excessive denoising and being unnatural [23, 24].

### 1.2. Soft Threshold Denoising

**Figure 3:**
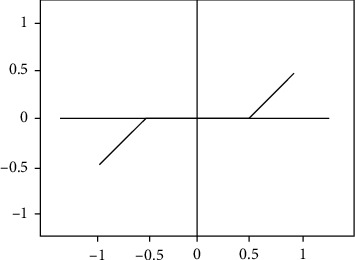
Soft threshold denoising.

As shown in Figure 3, the soft threshold denoising is that after the wavelet coefficient is decomposed, shrink the wavelet coefficients whose absolute value is greater than *T* to the zero point, and set the wavelet coefficient whose absolute value is smaller than *T* to zero. Set the threshold *T* to 0.5. The specific expression is as follows [22]:

(2) $$\begin{array}{c} \rho_{T}\left(x\right)=\left\{\begin{array}{cc} x-T, & x\geq T,\\ x+T, & x\leq -T,\\ 0, & \left|x\right|\leq T. \end{array}\right. \end{array}$$

In this way, the coefficient change is more natural and softer than the hard threshold denoising [25, 26].

## 2. Materials and Methods

### 2.1. Calculation Method of OEF

In this paper, the two-chamber model [12, 27] is used to calculate the relevant parameters, and the OEF value is finally calculated. The two-chamber model treats the magnetized material and the tissue of the magnetized material as two chambers. In MRI analysis of cerebral blood vessels, magnetized substance refers to hemoglobin in the capillaries and blood vessels; the tissue of the magnetized substance is brain substance. In the BOLD effect fMRI image, the brain parenchyma corresponding to a pixel contains many capillaries. Assuming that the distribution direction of capillaries in the brain parenchyma is random, its magnetic moment can be decomposed into short-term scale $\overset{¯}{S_{s}}\left(t\right)$ and long-term scale $\overset{¯}{S_{l}}\left(t\right)$:

(3) $$\begin{array}{c} \bar{S_{s}}\left(t\right)=\rho \cdot \left(1-\lambda \right)\cdot \exp\left[-0.3\cdot \varsigma \cdot {\left(\delta \bar{w}\cdot t\right)}^{2}\right], \end{array}$$

(4) $$\begin{array}{c} \bar{S_{l}}\left(t\right)=\rho \cdot \left(1-\lambda \right)\cdot \exp\left[-R2_{c}^{\prime }\cdot \left|t-t_{c}\right|\right], \end{array}$$

where $t_{c}={\left(\delta \bar{w}\right)}^{-1}$, $R2_{c}^{\prime }=\lambda \cdot \delta \bar{w}$, *ς* is a constant, *ρ* is a constant, and *λ* is the relative volume of the magnetized material. $\delta \bar{w}$ in equation (3) can be obtained from

(5) $$\begin{array}{c} \delta \bar{w}=\gamma \cdot \frac{4}{3}\cdot \pi \cdot \Delta \chi \cdot \text{Hct}\cdot \text{OEF}\cdot B_{0}. \end{array}$$

Among them, Hct is the hematocrit, *B*_0_ is the intensity of the external magnetic field, Δ*χ* is the magnetic susceptibility shift between deoxyhemoglobin and oxyhemoglobin, usually 0.18 ppm/Hct, *γ* is the magnetic susceptibility of the substance, and $\delta \bar{w}$ is the frequency deviation of the magnetized substance. Calculate *λ* by short-term scale fitting of the brightness change curve in the image and then substitute *λ* into equation (4) to calculate $\delta \bar{w}$ by long-term scale fitting, and finally, substitute $\delta \bar{w}$ into equation (5) to calculate the OEF value [28].

### 2.2. Adaptive Wavelet Threshold Denoising Method Based on Bayesian Estimation

Research on the denoising method of wavelet threshold has been active recently. Many new wavelet threshold denoising methods have been derived for the improvement of soft and hard thresholds and the different calculation methods of thresholds. Based on the Bayesian estimation, LkahwinderKaur proposed the NormalShrink wavelet threshold denoising method for the problem that the Donoho threshold method [29] cannot maximize the separation of image and noise at each level of wavelet. However, this method has a disadvantage; the image after denoising loses some details, and the edges appear blurry. In this paper, the wavelet threshold denoising method of NormalShrink is further improved. A wavelet adaptive threshold method based on Bayesian estimation [30] is used to denoise the image. After wavelet decomposition, according to different wavelet subband characteristics, the improved wavelet scale function parameter equations are used to determine the optimal threshold *T* that is appropriate for each wavelet decomposition scale.

In the process of denoising the image, if the prior information of the image can be considered and then the optimal threshold is obtained for the risk function, the result error will be reduced. The coefficients of each subband of image wavelet decomposition are basically symmetrically distributed near the zero point, forming a spike distribution, which can be described by a zero-mean generalized Gauss distribution GGD.

The general Gauss distribution is [31, 32]

(6) $$\begin{array}{c} {\text{GG}}_{\sigma_{X},\beta }\left(x\right)=C\left(\sigma_{X},\beta \right)\exp\left\{-{\left[\alpha \left(\sigma_{X},\beta \right)\;|\;x\;|\;\right]}^{\beta }\right\}-\infty <x<\infty ,\sigma_{X}>0,\beta >0,\\ \alpha \left(\sigma_{X},\beta \right)=\sigma_{X}^{-1}{\left[\frac{\Gamma \left(3/\beta \right)}{\Gamma \left(1/\beta \right)}\right]}^{1/2},\\ C\left(\sigma_{X},\beta \right)=\frac{\beta \alpha \left(\sigma_{X},\beta \right)}{2\Gamma \left(1/\beta \right)},\\ \Gamma \left(t\right)=\int_{0}^{\infty }e^{-u}u^{t-1}du, \end{array}$$

where *σ*_*X*_ is the standard deviation and *β* is the morphological parameter.

Suppose that *X* obeys a mean of zero and the variance is a Gaussian distribution of *σ*_*X*_^2^, that is, *X* ~ *N*(0, *σ*_*X*_^2^), *β* = 2, then the Bayesian risk estimation function is

(7) $$\begin{array}{c} \gamma \left(T\right)=E{\left(\overset{\wedge }{X}-X\right)}^{2}=E_{X}E_{Y\;|\;X}{\left(\overset{\wedge }{X}-X\right)}^{2},\\ \overset{\wedge }{X}=\eta T\left(Y\right),Y\;|\;X∼N\left(x,\sigma^{2}\right),X∼{\text{GG}}_{\sigma_{X},\beta }. \end{array}$$

For a given set of parameters, the threshold *T* is searched so that the result of *γ*(*T*) in (4) is the minimum. $T^{*}\left(\sigma_{X},\beta \right)=\arg\underset{T}{\min r\left(T\right)}$ is used to represent the optimized threshold function.

Next, the optimization threshold *T*^∗^ is obtained:

(8) $$\begin{array}{c} E_{X}E_{Y\;|\;X}{\left(\overset{\Lambda }{X}-X\right)}^{2}=\int_{-\infty }^{+\infty }\int_{-\infty }^{+\infty }{\left(\eta_{T}\left(y\right)-x\right)}^{2}p\left(y\;|\;x\right)p\left(x\right)dydx=\sigma^{2}\omega \left(\frac{\sigma_{X}^{2}}{\sigma^{2}},\frac{T}{\sigma }\right),\\ \omega \left(\sigma_{X}^{2},T\right)=\sigma_{X}^{2}+2\left(T^{2}+1-\sigma_{X}^{2}\right)\overset{-}{\phi }\left(\frac{T}{\sqrt{1+\sigma_{X}^{2}}}\right)-2T\left(1+\sigma_{X}^{2}\right)\phi \left(T,1+\sigma_{X}^{2}\right). \end{array}$$

The standard density function is

(9) $$\begin{array}{c} \phi \left(x,\sigma^{2}\right)=\left(\frac{1}{\sqrt{2{\pi \sigma }^{2}}}\right)\exp\left(-\left(\frac{x^{2}}{2\sigma^{2}}\right)\right),\\ \overset{-}{\phi }\left(x\right)=\int_{x}^{\infty }\phi \left(t,1\right)dt. \end{array}$$

So

(10) $$\begin{array}{c} T_{B}\left(\sigma_{X}\right)=\frac{\sigma^{2}}{\sigma_{X}}. \end{array}$$

*T*_*B*_(*σ*_*X*_) is an approximation of *T*^∗^(*σ*_*X*_, 2), and its maximum deviation is not more than 0.01. The noise variance *σ*^2^ in (10) can be estimated by using the median absolute variance for the highest frequency subband [28]:

(11) $$\begin{array}{c} {\overset{\Lambda }{\sigma }}^{2}={\left[\frac{\text{Median}\left|Y_{i,j}\right|}{0.6745}\right]}^{2}Y_{i,j}\in \text{subband }{HH}_{1}, \end{array}$$

(12) $$\begin{array}{c} {\overset{\Lambda }{\sigma }}_{Y}^{2}=n^{-2}\overset{n}{\underset{i,i=1}{\sum }}Y_{ij}^{2}\left(n\times n\text{ is the considered subband size}\right), \end{array}$$

Finally, a data-driven, subband-based adaptive threshold is obtained as shown in [33]

(13) $$\begin{array}{c} {\overset{\Lambda }{T}}_{B}\left(\overset{\Lambda }{\sigma_{X}}\right)=\frac{{\overset{\Lambda }{\sigma }}^{2}}{\overset{\Lambda }{\sigma_{X}}}. \end{array}$$

## 3. Experimental Results

### 3.1. Experimental Method

There were 12 young normal volunteers in this experiment, all aged 22-30 years old, with no gender selectivity. The 12 normal volunteers were scanned with GESSE sequence of brain parenchyma, and the scanning level was located above the lateral ventricle. Scanning parameters are as follows: tr = 1.5 s, TE = 56 ms, bandwidth 62.5 kHz, image matrix 256∗256, gradient echo number 32, echo gap 1.5 ms, and layer thickness 7.5 mm. 32 DICOM images obtained from the GESSE sequence are used as the original data input.

### 3.2. Wavelet Threshold Denoising Method

In this paper, three evaluation indexes of image display, SNR (signal-to-noise ratio), and MSE (mean square error) are considered in the comparison of various denoising methods. Each indicator verifies one of the advantages and disadvantages of the denoising method. Among them, the image display shows that the denoising image can be visually observed; the signal-to-noise ratio is aimed at retaining the signal while suppressing how much noise; the mean square error is suitable for expressing the sharpness of a picture.

#### 3.2.1. SNR (Signal-to-Noise Ratio)

SNR (signal-to-noise ratio) is one of the more traditional methods for measuring the amount of noise in a signal [34, 35]. It is often used as an indicator for denoising effect evaluation. The unit is decibel (dB), which is defined as

(14) $$\begin{array}{c} \text{SNR}=10\mathrm{lg}\left(\frac{\left(1/MN\right)\sum_{i=0}^{M-1}\sum_{j=0}^{N-1}f_{i,j}^{2}}{\text{MSE}}\right). \end{array}$$

We can know that the larger SNR is after denoising, the better the denoising effect will be.

#### 3.2.2. MSE (Mean Square Error)

The MSE (mean square error) shows the sharpness of the image [36] and the root mean square error between the original signal A and the denoised estimated signal B is defined as

(15) $$\begin{array}{c} \text{MSE}=\frac{1}{MN}\overset{M-1}{\underset{i=0}{\sum }}\overset{N-1}{\underset{j=0}{\sum }}{\left(\bar{f_{i,j}}-f_{i,j}\right)}^{2}. \end{array}$$

We can know that the smaller MSE is after denoising, the better the denoising effect will be.

### 3.3. Generation of Rician Noise

Rician noise is not additive, but data dependent. First, a noiseless MR image *A* is defined on a discrete grid *I*, *A* = {*a*_*i*_ | *i* ∈ *I*}, and a set of random numbers is used as the brightness of image *A*. Taking *σ* as the standard deviation of Gaussian noise, two sets of Gaussian random numbers *X* = {*x*_*i*_ | *i* ∈ *I*} and *Y* = {*y*_*i*_ | *i* ∈ *I*} are formed, and the average of the two sets of numbers is 0 and has the same standard deviation *σ*. Then, the following *M* = {*m*_*i*_ | *i* ∈ *I*} is the Rician distribution:

(16) $$\begin{array}{c} m_{i}=\sqrt{{\left(a_{i}+x_{i}\right)}^{2}+y_{i}^{2}}. \end{array}$$

### 3.4. Simulation Experiment Process and Result Analysis

#### 3.4.1. Simulation Experiment Process

Three methods of Gaussian filtering and wavelet transform (traditional hard threshold, traditional soft threshold denoising, and adaptive threshold denoising based on Bayesian estimation) are used to denoise the signals with different standard deviations *σ* of Rician noise. By observing the simulation results, the advantages and disadvantages of Gaussian filtering and traditional wavelet threshold denoising were compared and verified, as well as the advantages and feasibility of the new Bayesian estimation adaptive threshold wavelet denoising in MRI image signal denoising.

#### 3.4.2. Generation of Rician Noise

**Figure 4:**
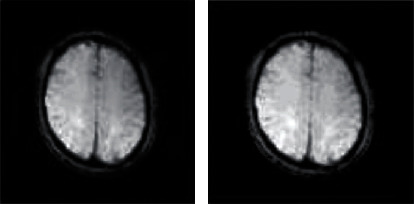
Original image and noisy image.

*(1) Load the Original MRI Signal*. A signal containing Rician noise is generated using MATLAB simulation software, as shown in Figure 4. The left picture shows the original fMRI signal image, and the right picture shows the noisy signal image with Rician noise. And the standard deviation of noise is 0.009. It can be observed that the noisy image is slightly blurred. Since the standard deviation of noise is small, no obvious blur phenomenon is observed.

**Figure 5:**
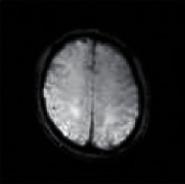
MRI image after Gaussian filtering.

*(2) Denoising Using Gaussian Filtering*. The noise image is denoised by Gaussian filtering, which uses a two-dimensional operator of size 5∗5. The image after denoising by Gaussian filtering is shown in Figure 5.

**Figure 6:**
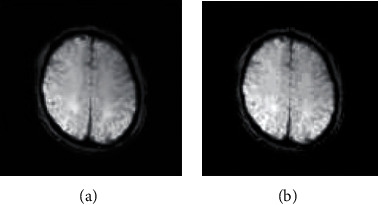
(a) Soft threshold denoised image. (b) Hard threshold denoised image.

*(3) Denoising Using Traditional Wavelet Hard and Soft Thresholds*. In the MATLAB simulation program, the original signal is decomposed by the “haar” wavelet function, and the signal is denoised by the soft threshold and the hard threshold, respectively. The result is shown in Figure 6.

*(4) Signal Processing Using the New Bayesian Estimation Adaptive Threshold Wavelet Denoising*.

*(5) Calculation of SNR and MSE*. The formulas of the SNR and the MSE are as shown in (14) and (15), and the SNR and MSE of the denoised image are calculated according to the formula.

#### 3.4.3. Analysis of Results

**Figure 7:**
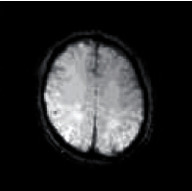
The new Bayesian estimation of adaptive threshold wavelet denoising image.

Firstly, by observing the noise reduction comparison diagram of Gaussian filtering and three kinds of wavelet transform (Figures 5–7), it can be seen that the signals obtained after noise reduction by wavelet transform have better similarity and smoothness. Compared with the traditional Gaussian filter denoising method, the signal denoising method based on wavelet transform can remove the noise in the signal more effectively.

The comparison of the advantages and disadvantages of each denoising method is shown in Tables 1 and 2:

Tables 1 and 2 show the SNR and root mean square error of the original signal after denoising the image with different standard deviation noises by Gauss filtering, traditional wavelet threshold transform, and new Bayesian estimation adaptive threshold wavelet transform. According to the evaluation criteria of denoising performance, the signal-to-noise ratio of the denoised signal by wavelet transform is higher than that of the denoised signal by Gaussian filter, and the root mean square error is lower. The data show that the denoised signal by wavelet transform has better similarity with the original signal, and the denoised signal by wavelet transform retains more energy of the original signal.

**Figure 8:**
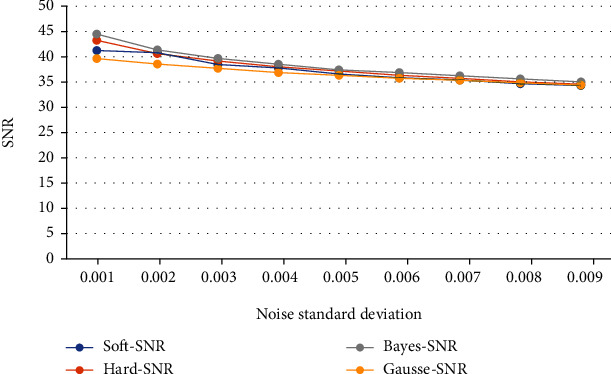
Comparison of SNR results of four denoising methods.

**Figure 9:**
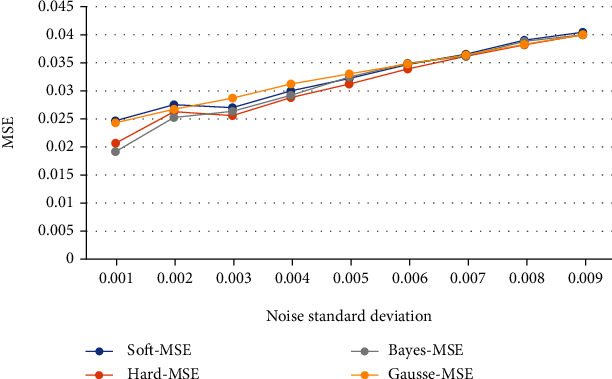
Comparison of MSE results of four denoising methods.

In order to make a more intuitive comparison, this paper makes a broken line chart based on the table data, as shown in Figures 8 and 9.

It can be concluded that Bayesian estimation of adaptive wavelet denoising is superior to other denoising methods, and its application to fMRI image denoising can improve the degree of signal noise separation and retain useful signals to the maximum extent.

### 3.5. Simulation Experiment

**Figure 10:**
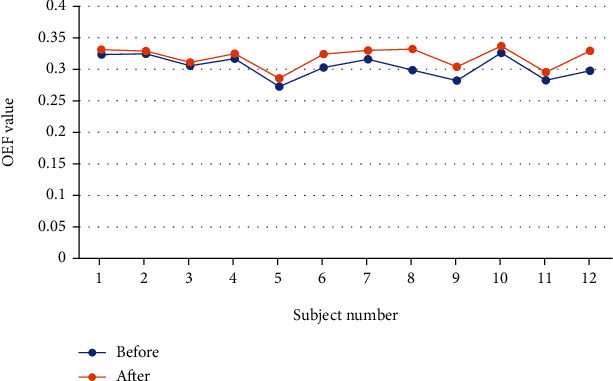
Comparison of OEF results before and after optimization.

The new adaptive threshold wavelet denoising method based on Bayesian estimation was applied to the actual experiment to try to optimize the results of mathematical modeling and measurement of OEF [15]. Firstly, the original postprocessing program was used to analyze the experimental data of 12 groups of volunteers, and the OEF results were obtained. Then, the optimized postprocessing program was used to analyze the experimental data of the volunteers to obtain new results. According to the gold standard measured by PET [37, 38], the two groups of results were compared and analyzed, as shown in Table 3 and Figure 10.

## 4. Conclusions

In the postprocessing process of OEF mathematical modeling, the adaptive threshold wavelet denoising method based on Bayesian estimation retained more useful signals than the original Gaussian filter. The 12 groups of optimized OEF results in the experiment all have a certain degree of increase compared to the OEF values before optimization, making the results closer to the gold standard value of 35% in PET measurement. The experiment preliminarily verified the feasibility and superiority of applying the adaptive threshold wavelet denoising method based on Bayesian estimation to fMRI signal processing and analysis.

## Figures and Tables

**Table 1:** SNR comparison of image signals after noise reduction.

	Soft-SNR	Hard-SNR	Bayes-SNR	Gausse-SNR
*σ* = 0.001	41.2198	43.2875	44.4607	39.6104
*σ* = 0.002	40.7725	40.6271	41.3334	38.5229
*σ* = 0.003	38.4023	39.1372	39.6665	37.7407
*σ* = 0.004	37.7854	37.9235	38.4900	36.8859
*σ* = 0.005	36.5943	37.1237	37.3624	36.2954
*σ* = 0.006	35.8307	36.2856	36.8062	35.7564
*σ* = 0.007	35.3401	35.6654	36.1744	35.3158
*σ* = 0.008	34.6803	35.1072	35.5330	34.8268
*σ* = 0.009	34.3478	34.6372	35.0086	34.4037

**Table 2:** MSE comparison of image signals after noise reduction.

	Soft-MSE	Hard-MSE	Bayes-MSE	Gausse-MSE
*σ* = 0.001	0.0246	0.0207	0.0192	0.0243
*σ* = 0.002	0.0275	0.0263	0.0253	0.0267
*σ* = 0.003	0.0270	0.0256	0.0263	0.0287
*σ* = 0.004	0.0300	0.0288	0.0292	0.0312
*σ* = 0.005	0.0322	0.0312	0.0325	0.0330
*σ* = 0.006	0.0347	0.0339	0.0349	0.0348
*σ* = 0.007	0.0365	0.0362	0.0362	0.0364
*σ* = 0.008	0.0390	0.0382	0.0388	0.0383
*σ* = 0.009	0.0404	0.0400	0.0400	0.0400

**Table 3:** Comparison of OEF results before and after optimization.

	1	2	3	4	5	6
Before	0.324 ± 0.035	0.325 ± 0.033	0.306 ± 0.043	0.317 ± 0.028	0.273 ± 0.023	0.303 ± 0.025
After	0.331 ± 0.049	0.329 ± 0.044	0.311 ± 0.044	0.325 ± 0.049	0.286 ± 0.036	0.324 ± 0.051
	7	8	9	10	11	12
Before	0.316 ± 0.0348	0.299 ± 0.022	0.282 ± 0.023	0.326 ± 0.028	0.283 ± 0.027	0.298 ± 0.044
After	0.330 ± 0.0518	0.332 ± 0.048	0.304 ± 0.074	0.337 ± 0.047	0.296 ± 0.050	0.329 ± 0.057

## Data Availability

The dataset that supports the findings and conclusion of this study are available from the corresponding author on reasonable request. The data are not publicly available due to privacy.
